# Analysis of Heart Rate Variability in Newborns Dogs with Different Types of Delivery during the First 35 Days of Life

**DOI:** 10.3390/vetsci11050225

**Published:** 2024-05-17

**Authors:** Jaqueline Valença Corrêa, Carolina Dragone Latini, Beatriz Almeida Santos, Amanda Sarita Cruz Aleixo, Keylla Helena Nobre Pacífico Pereira, Miriam Harumi Tsunemi, Luiz Henrique de Araujo Machado, Maria Lucia Gomes Lourenço

**Affiliations:** 1 Department of Veterinary Clinics, School of Veterinary Medicine and Animal Science, São Paulo State University (Unesp), Botucatu 18618-681, SP, Brazil; jaqueline.v.correa@unesp.br (J.V.C.); carolina.latini@unesp.br (C.D.L.); biasantos.vet@gmail.com (B.A.S.); amanda.cruz21@hotmail.com (A.S.C.A.); keylla.pacifico@unesp.br (K.H.N.P.P.); henrique.machado@unesp.br (L.H.d.A.M.); 2 Department of Veterinary Medicine, Federal University of Alagoas (UFAL), Viçosa 36570-900, AL, Brazil; 3 Department of Biostatistics, Institute of Bioscienses, São Paulo State University (UNESP), Botucatu 18618-689, SP, Brazil; m.tsunemi@unesp.br

**Keywords:** neonatology, dogs, newborns, electrocardiogram, heart rate variability

## Abstract

**Simple Summary:**

The present study aimed to evaluate the influence of the autonomic nervous system on cardiovascular function during the first 35 days of life in different types of delivery, using heart rate variability indices. Thirty newborns were equally divided into two groups based on delivery type: eutocic delivery and emergency cesarean section. Electrocardiographic evaluation was performed at birth (T0), 24 h postpartum (T1), and at 7, 14, 21, 28, and 35 days of life (T2 to T6). Physical parameters, neonatal reflexes, and Apgar scores were recorded. Over 35 days, newborns from the emergency cesarean section group presented lower heart rate variability than those born from eutocic delivery group, it was shown that the type of delivery should be considered for the assessment of autonomic nervous system activity in neonates. Thus, as predictive factors of vitality, heart rate variability and Apgar scores can help in the face of neonatal depression, demonstrating that delivery by emergency cesarean section can predispose newborns to delays in the autonomic influence on the heart.

**Abstract:**

The present study aimed to evaluate the influence of the autonomic nervous system on cardiovascular function during the first 35 days of life in different types of delivery, using heart rate variability (HRV) indices. Thirty newborns were equally divided into two groups based on delivery type: eutocic delivery (EG) and emergency cesarean section (CG). Electrocardiographic evaluation was performed at birth (T0), 24 h postpartum (T1), and at 7, 14, 21, 28, and 35 days of life (T2 to T6). Physical parameters, neonatal reflexes, and Apgar scores were recorded. Over 35 days, the values of the time domain indices were higher in the GE group and increased with age. In the frequency domain, the low frequency (LF) index was higher in the CG, and the opposite occurred for the high frequency (HF) index. Since the CG presented lower HRV than the EG, it was shown that the type of delivery should be considered for the assessment of autonomic nervous system activity in neonates. Thus, as predictive factors of vitality, HRV and Apgar scores can help in the face of neonatal depression, demonstrating that delivery by emergency cesarean section can predispose newborns to delays in the autonomic influence on the heart.

## 1. Introduction

The autonomic nervous system (ANS) plays an important role in modulating the cardiovascular system through sympathetic and parasympathetic components. One of the methods used to evaluate the effect of the ANS on the heart is the analysis of heart rate variability (HRV), which describes the oscillations of the intervals between consecutive heartbeats (R-R intervals) [1]. Heart rate variability is determined by measuring the time interval between two consecutive QRS complexes [2]. The fetal-neonatal transition is characterized by a critical period, given the physiological adaptations that occur in neonates. Soon after birth, the newborn’s body must take over the organic functions that were previously performed by the placenta. Changes in endocrine, respiratory, and cardiovascular patterns assist in this transition [3,4]. At birth, the inspiratory reflex is triggered by the increased partial pressure of carbon dioxide in the umbilical vessels and the drop in body temperature, increasing peripheral vascular resistance and reducing pulmonary vascular resistance [3,5,6]. Pulmonary expansion triggers the release of prostacyclin and nitric oxide, increasing vasodilation and blood flow [3,5].

At this stage, there is greater susceptibility to hypoxia due to the high metabolic demand, immaturity of the chemoreceptors present in the carotid sinus and incomplete development of respiratory function [3,7]. During birth, newborns can present with physiological hypoxemia due to the fetal asphyxia transition, which occurs simultaneously with uterine contractions [8]. Factors that prolong neonatal asphyxia to a permanent state, including emergency cesarean section due to dystocia, prolonged labor, and anesthetic agents used in cesarean sections, can promote respiratory impairment, leading to a greater risk of death [9,10].

As the autonomic nervous system does not completely develop during the neonatal period, the investigation of the evolution of neural modulation during this phase, as well as the evaluation of HRV in the different periods of gestation and development, shows great importance, as HRV can be used as a prognostic factor of diseases [11], both in human medicine [12,13,14,15] and veterinary medicine [16].

The mortality rates due to dystocia and cesarean section in newborns are high [17]. The mortality rate of animals born by cesarean section is between 4 and 15% [18,19,20,21,22], and there is a difference in the number of neonates that die from cesarean section (8% at birth and 13% after 2 h) compared to those that die from eutocic delivery (2.2% at birth and 8% after 24 h) [23]. Resuscitative interventions are often needed in cases of dystocia and long labor. Even with the appropriate use of anesthetic protocols, newborns will still exhibit some degree of respiratory depression [24].

As demonstrated by Vassalo [25], in the evaluation of neonatal viability in dogs with eutocic and cesarean deliveries, newborns with eutocic delivery presented higher Apgar scores (7.6 ± 0.3) and neonatal reflexes (4.6 ± 0.2) than newborns delivered by cesarean section (4.3 ± 0.3; 1.7 ± 0.2). Newborns delivered by cesarean section had greater respiratory depression and low gas exchange efficiency during their first inspirations, triggering severe hypoxia and central cyanosis [26].

Currently, there are few studies in canine species on the development of HRV in the neonatal phase, as well as its correlation with different types of delivery, considering prolonged hypoxemia caused by emergency cesarean section. Thus, the present study aimed to evaluate how ANS activity influences the cardiac activity of neonatal dogs with different types of delivery throughout the first 35 days of life. This evaluation was performed by analyzing HRV indices, correlating these data with vitality at birth (Apgar score) obtained for both types of delivery.

## 2. Materials and Methods

### 2.1. Local

The animals were cared for in the Small Animal Clinic, Reproduction Service of the Veterinary Hospital of FMVZ Unesp and at the owners’ homes in 2020 and 2021. The study was performed after approval by the Ethics Committee on Animal Use of the Faculty of Veterinary Medicine of Unesp (CEUA)—FMVZ—Botucatu (protocol no. 0115/2020) and upon obtaining the owners’ signatures on the informed consent form.

### 2.2. Pregnant Females

We included pregnant females of different breeds of the canine species aged between 1 and 10 years who had been vaccinated and dewormed. In both groups, a physical examination of the pregnant females was performed, with data on the following parameters collected: mucosal coloration, heart rate (HR), respiratory rate (RR), body temperature (T°C), auscultation and abdominal palpation. The bitches evaluated did not present any type of systemic comorbidity.

Eutocic deliveries were considered those in which the offspring were expelled spontaneously, without any kind of obstetric maneuver. The pregnant females were monitored during fetal expulsion with the release of the fetal membranes and cleaning and rupture of the umbilical cord. The remaining assessments of the offspring were performed at home.

Emergency cesarean deliveries were those in which there were signs of dystocia, making surgical intervention necessary. Dystocia was defined as poor positioning or fetal development, very large fetuses, a narrow pelvic canal, uterine inertia, fetal putrefaction, a long interval between births, endotoxemia in the pregnant female or fetal distress assessed by ultrasonography. Gestational data (age, primiparity, causes of dystocia and drug administration) were recorded.

### 2.3. Anesthetic and Surgical Procedure

For cesarean section, the anesthetic protocol instituted for the pregnant females was induction with intravenous propofol at a dose for loss of the laryngotracheal reflex and epidural anesthesia with 2% lidocaine at a dose of 1.0 mL for every 5 kg. Isoflurane was diluted in 100% oxygen in the valved circular anesthetic circuit for anesthetic maintenance. After removal of the fetuses, fentanyl at a dose of 5 mg/kg was administered intravenously and slowly to the pregnant females.

After induction of anesthesia, the females underwent extensive trichotomy in the region of the abdomen and antisepsis. An incision was made in the preretroumbilical region at the linea alba with exposure of the uterine horns. The fetuses were carefully removed and separated from the placenta, the umbilical cords were sectioned and clamped with hemostatic forceps, and the fetuses received neonatal care.

### 2.4. Neonatal Resuscitation

All neonates delivered by emergency cesarean section were resuscitated according to the protocol described by Pereira et al. (2022) [24]. Immediately after removal of the neonate from the uterus, the surgeon removed the fetal wrappings to keep the airway clear, and fluids were flushed out of the nostrils and oral cavity with the help of a compress or warm towel.

The newborns were taken to a warm place, and gentle aspiration of nasal secretions and the oral cavity was performed with the help of a pediatric nasal aspirator. Tactile stimulation was initiated by vigorous massage with a heated compress, rubbing the back, chest, genital and umbilical regions with the intention of drying and stimulating breathing. Massage was continuous until breathing was perceived.

If a low neonatal vitality score (modified Apgar score and neonatal reflexes), respiratory distress and/or neonatal bradycardia (HR < 180 bpm) was observed in pups delivered by cesarean section, we initiated resuscitation procedures.

A Jen Chung or Governor Vessel 26 acupuncture point was used, and a 26-gauge needle was inserted in the midline of the base of the nostril, rotating clockwise. Respiratory support was provided with constant oxygen flow by using a mask appropriate for the size of the newborn. Aminophylline at a dose of 0.2 mL per 100 g of weight was administered sublingually at a concentration of 24 mg/mL.

### 2.5. Animals

In both groups, information such as breed, age, weight, HR, RR, T°C, neonatal reflexes (sucking, rooting and straightening reflexes), Apgar score (between one and five minutes after birth as proposed by Veronesi [20]) and electrocardiogram (ECG) results were recorded at birth (T0). Data on physical parameters (HR, RR, T°C), neonatal weighing and ECG results were collected 24 h after birth (T1) and consecutively once per week until 35 days of age (7 days—T2, 14 days—T3, 21 days—T4, 28 days—T5 and 35 days—T6), for a total of seven times.

Newborns in the eutocic group (EG) were included when they met at least three of the following criteria: an HR greater than 180 beats per minute (bpm), reddened mucous membranes, an RR greater than 15 movements per minute (mpm), and an Apgar score greater than 7 at assessment.

Neonates in the emergency cesarean section group (CG) were included when they met at least three of the following criteria: an HR less than 180 bpm (indicating neonatal bradycardia), cyanotic mucous membranes, an RR less than 15 mpm, an Apgar score less than 7, and the presence of meconium in the amniotic fluid and/or on fetus.

The exclusion criteria were neonates with any congenital malformation and neonatal death before 35 days of life.

### 2.6. Apgar Score

At birth, for the evaluation of vitality and viability, the modified Apgar score for dogs at the immediate moment of birth (in the first five minutes after birth) was used. The parameters evaluated were HR, RR, reflex irritability, muscle tone, and mucosal coloration. Reflex irritability was assessed by a painful stimulus in the interdigital space and muscle tone was evaluated by keeping the newborn in a supine position and observing limb movement. Each parameter was scored from 0 to 2, and the sum of all parameters resulted in the Apgar score, as proposed by Veronesi [20] in Table 1 below.

### 2.7. Neonatal Reflexes and Neonatal Vitality

**Figure 1:**
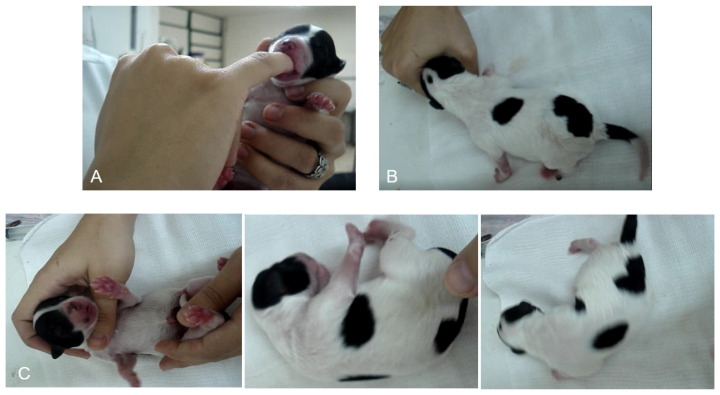
Demonstration of suckling (**A**), rooting (**B**) and righting reflexes (**C**) in a neonate.

Neonatal reflexes were assessed between one and five minutes after birth as reported by Vassalo [27] as follows: the suckling reflex was evaluated by the examiner introducing their little finger into the mouth of the newborn and observing the strength of suction. Straightening reflexes were evaluated by placing the animal in dorsal recumbency above a soft and warm surface and waiting for the straightening of the body with a rapid return to ventral recumbency. The search reflex was evaluated by placing the animal’s face near a circle made with the hand and waiting for the newborn to place its face in the circle automatically. Each reflex received a score of 0 (no response), 1 (weak), or 2 (strong). The sum of the scores demonstrated neonatal vitality, which was classified as follows as proposed by Vassalo [27]: 0–2, weak vitality; 3–4, moderate vitality; and 5–6, normal vitality in the Table 2 and Figure 1 below.

### 2.8. Electrocardiogram

The electrocardiogram was performed using a computerized device (ECGPC—TEB—Digital Electrocardiograph), recorded in bipolar leads (I, II and III) and increased unipolar leads (aVR, aVL and aVF) in the frontal plane as standardized by Tilley et al. (1992) [28] and reviewed by Santilli et al. (2020) [2]. The animals were positioned in the right lateral recumbency position on a surface covered with a rubber floor plate to avoid interference in the electrocardiographic tracing. The electrodes were moistened with alcohol and fixed on the thoracic limbs near the olecranon in the caudal aspect and on the pelvic limbs over the patellar ligament in the cranial aspect. The animals evaluated were submitted to this procedure without any type of sedation, tranquilizer, or anesthesia.

The electrocardiographic tracings were recorded by using software for HRV analysis (Kubios 3.1 Software—Biomedical Signal Analysis Group, Department of Applied Physics, **University of Eastern Finland, Kuopio**, Finland). Heart rate variability was assessed by measuring successive periods of electrocardiographic tracing, excluding preterm beats and the subsequent compensatory beats.

#### Heart Rate Variability Analysis

Heart rate variability indices were analyzed in the time and frequency domains. In the time domain, the standard deviation of the mean of all R-R intervals (SDNN; expressed in ms), the square root of the root mean square of the differences between R-R intervals (RMSSD; expressed in ms), and the percentage of adjacent R-R intervals with a difference in duration greater than 50 ms (pNN50) were evaluated. The SDNN was used to reflect all cyclic components of variability in the series of recorded R-R intervals. The RMSSD was used as an estimate of the high-frequency variations in the short-term recordings of the R-R intervals. The parameters in the frequency domain were the low frequency (LF) measured in normalized units (NU) ranging from 0.04 to 0.15 Hz, the high frequency (HF) measured in NU with a range of 0.15 to 0.4 Hz, and the LF/HF ratio. The LF was used to examine sympathetic modulations, while the HF was used to evaluate parasympathetic modulations. Finally, the LF/HF ratio was used to assess sympathovagal balance (Table 3).

## 3. Statistical Analysis

For statistical analysis, variables with repeated measures over time were used and submitted to the Shapiro-Wilk normality test. For comparison between the EG and CG, the *t* test for independent samples or the Mann-Whitney test was performed according to the normality assumption.

Differences between times within each group were determined by Friedman’s test at the 5% significance level. Pearson and Spearman correlations were determined for HRV, T°C and the Apgar score. All tests were performed at the 5% level of significance (*p* < 0.05).

## 4. Results

### 4.1. Neonates and Apgar Score

A total of 42 neonates were evaluated in this study: 18 animals in the EG and 24 in the CG. Three neonates from the EG and nine from the CG were excluded due to neonatal death. In the eutocic group, 86% (13/15) of the dogs were Spitz Germanes, and 13.3% (2/15) were mongrels; among these, 13.3% (2/15) were females, and 86.6% were males (13/15). In the emergency cesarean section group, the predominant breed was the French Bulldog (80%; 12/15), followed by American Bully (13.3%; 2/15) and Dachshund (6.6%; 1/15), and a total of 60% (9/15) were female and 40% (6/15) were male.

Based on the neonatal reflex score, the CG (2.4 ± 2.0) showed lower vitality than the EG (5.4 ± 0.6), presenting a significant difference (*p* = 0.0003).

The pregnant females in the EG were multiparous (3/4), weighed 3.1 ± 0.8 kg and were aged between 2 and 6 years (3.5 ± 0.9 years). The average number of pups per birth was approximately 4 to 5 (4.5 ± 2.6 pups). Among all pups born, there was only one stillbirth (1/18) and 2 neonatal deaths (2/18).

On the other hand, females in the CG were primiparous (5/5), with a body weight of 12.9 ± 1.7 kg and an age between 1 and 10 years (3.2 ± 3.8 years). The average number of pups per birth was 4 (4.2 ± 2.1 pups). However, 1 mummified fetus (1/24), 3 stillbirths (3/24) and 5 neonatal deaths (5/24) were recorded in this group. All females underwent emergency cesarean section due to uterine inertia.

### 4.2. Eutocic Group

In the evaluation of the physiological parameters shown in Table 4, the EG showed a significant difference (*p* < 0.05) in HR between the first moments (T0, T1, T2) when compared to Day 21 of life, with an increase in this parameter with advancing age. However, the heart rate on Days 28 and 35 showed a significant decrease compared to Day 21.

In the eutocic group, as observed in Table 4, T°C increased from the 3rd week of life, differing from that in the initial time points of the study (T0 and T1). The other time points (T2 and T3) also showed significant differences compared to the final time points (T5 and T6).

**Figure 2:**
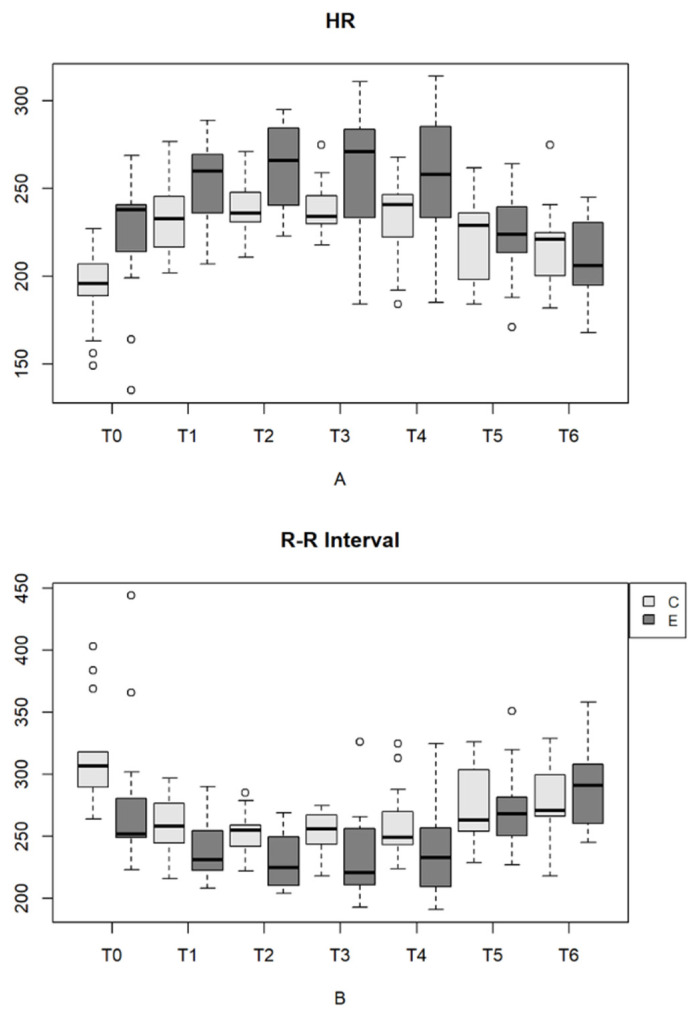
(**A**)—HR variation (bpm) in newborn dogs at each type of delivery and time point. (**B**)—R-R interval (ms) in newborn dogs at each type of delivery and time point. Parturition E (eutocic); Parturition C (emergency cesarean). **Outliers are represented by circles**.

In the evaluation of HR on electrocardiogram, as shown in Table 5 and Figure 2, both HR and the R-R interval showed significant differences (*p* < 0.05) between the initial time points (T2, T3 and T4) and the last time points (T5 and T6). Similar to heart rate shown on auscultation, HR shown on ECG increased with age, with a decrease in the parameter from Day 21 onward.

**Figure 3:**
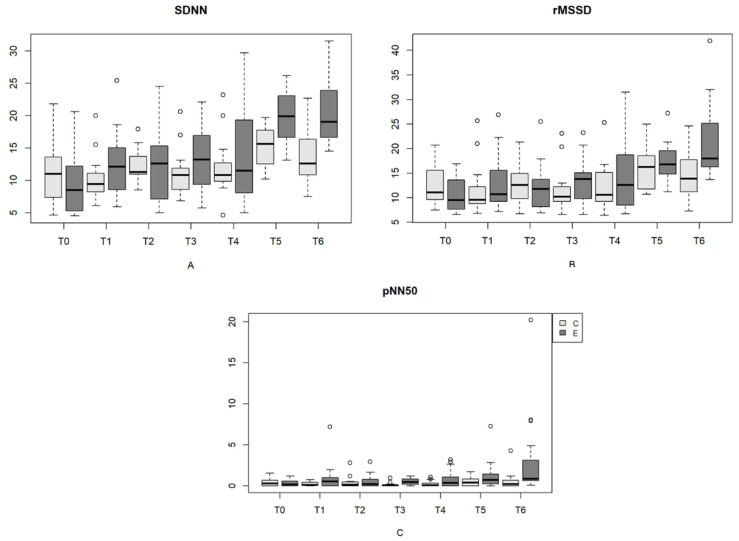
(**A**)—SDNN variation (ms) in newborn dogs at each type of delivery and time point. (**B**)—Variation in rMSSD (ms) in neonatal dogs at each type of delivery and time point. (**C**)—Variation in pNN50 (%) in newborn dogs at each type of delivery and time point. Parturition E (eutocic); Parturition C (emergency cesarean). **Outliers are represented by circles**.

Similarly, in the assessment of HRV in the time domain indices between the time points of delivery in the EG, in the analysis of the SDNN, there was a significant difference (*p* < 0.05) between T0, T1, T2 and T4 and the last time points (T5 and T6). In turn, the RMSSD showed a significant difference (*p* < 0.05) only at birth and from Day 7 to Day 35. The pNN50 did not differ (*p* > 0.05) at any evaluated times. In all these indices (SDNN, RMSSD and pNN50), there was an increase in the means with advancing age, as shown in Table 6 and Figure 3.

In the analysis of the frequency domain indices, shown in Table 7, the HF and the LF/HF ratio differed (*p* < 0.05) between Day 1 of life and Day 28, while the LF showed no significant difference (*p* > 0.05). In the present study, there was an increase in LF and the LF/HF ratio with advancing age. In turn, HF increased from birth (T0) to Day 1, with a decrease in this index until Day 28, which increased again on Day 35.

### 4.3. Cesarean Group

In the analysis of HR in the CG, there was a significant difference (*p* < 0.05) only between birth (T0) and the other time points (T1, T2, T3, T4, T5 and T6) because the newborns had neonatal bradycardia at the time immediately after birth. This parameter also differed between Day 7 (T2) and Day 35 (T6). In this group, there was an increase in HR until Day 7 and a decline in heart rate on Days 28 and 35. On the other hand, the RR also showed an increase, with significant differences (*p* < 0.05) between the initial time points (T0 and T1) and the last time points (T5 and T6).

In the evaluation of T°C and newborn weight, this group showed a significant difference (*p* < 0.05) among the time points. For both parameters, this difference started from Day 14, with increasing values as age increased, as shown in Table 4.

The heart rate and the R-R interval, obtained by ECG, differed (*p* < 0.05) between birth and the subsequent time points (T1, T2, T3 and T4) and the 21st day of life, with an increase in HR and a decrease in the R-R interval (Table 5 and Figure 2).

**Figure 4:**
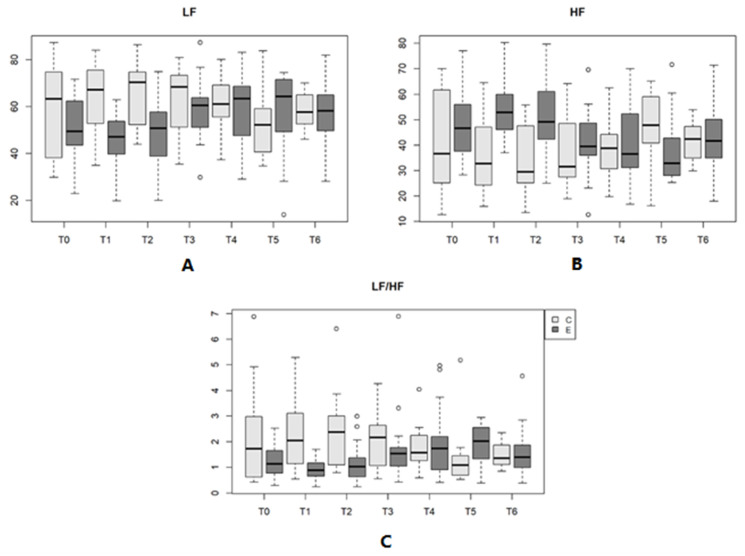
(**A**)—LF variation (n.u.) in newborn dogs at each type of delivery and time point. (**B**)—HF variation (n.u.) in newborn dogs at each type of delivery and time point. (**C**)—Variation in the LF/HF ratio in newborn dogs at each type of delivery and time point. Parturition E (eutocic); Part C (emergency cesarean). **Outliers are represented by circles**.

In the analysis of HRV in the time domain, the SDNN and RMSSD increased across the 35 days, with a significant difference (*p* < 0.05) only between Day 1 and Day 28. Similar to the eutocic group, in the CG, an increase in the mean values of these indices was observed across the 35 days (Table 6 and Figure 4). In the frequency domain, there was no significant difference (*p* > 0.05) in any of the indices (the LF, HF and LF/HF ratio) among the time points evaluated (Table 7 and Figure 4).

The results, reported as the mean and standard deviation of the physiological parameters evaluated (HR, RR, T°C) and the body weights of the newborns in the eutocic and cesarean groups, with statistical significance between time points and groups are presented in Table 4.

The results, reported as the mean and standard deviation of HRV indices of the newborns in the EG and CG in the time (mean HR, mean R-R interval, SDNN, RMSSD and pNN50) and frequency domains (the LF, HF and LF/HF ratio), with statistical significance between time points and groups are shown in Table 5, Table 6, Table 7 and Figure 2, Figure 3, Figure 4.

### 4.4. Comparative Evaluation between the EG and CG

In the electrocardiography study, there was a significant difference (*p* < 0.05) in the HR and R-R interval from birth to Day 14, with a higher mean HR in the EG. In the time domain, the SDNN showed a significant difference (*p* < 0.05) on Days 28 and 35, the RMSSD showed a significant difference on Day 35, and the pNN50 showed a significant difference on Days 14 and 35 between groups. In most of the evaluations, the means of the EG were higher than those of the CG. In the frequency domain indices, there was a significant difference (*p* < 0.05) between groups in the LF on Days 1 and 7, the HF, and the LF/HF ratio on Days 1, 7, and 28. The cesarean group presented higher means in the LF and in the LF/HF ratio, while the EG means were higher for the HF at most of the evaluated time points.

In the frequency domain, on Day 1 of life, there was a negative correlation between the LF band and the LF/HF ratio versus the Apgar score (Rs = 0.46), so there was a positive correlation of the HF with the Apgar score (Rs = 0.46). On Day 7 of life, again, there was a negative correlation between the LF band (Rs = 0.38) and the LF/HF ratio 54 (Rs = 0.37) and the reverse was observed for the HF band (Rs = 0.37), as shown in Table 8.

#### Correlation among HRV Indices, Apgar Score and Neonatal Reflexes

In the correlation of the Apgar score with the HR and HRV indices, positive and negative correlations (*p* < 0.05) were observed in both groups at various times.

At birth, on the 7th and 14th days, there was a positive correlation of the HR and a negative correlation of the R-R interval (Rs = 0.39; 0.37; 0.43, respectively) with the Apgar score, as observed in Table 9. In the evaluation of HRV indices in the time domain, as shown in Table 8, there was a positive correlation (*p* < 0.05) between the Apgar score and SDNN on the 28th (Rs = 0.44) and 35th days (Rs = 0.49). The pNN50 showed a positive correlation (*p* < 0.05) with the Apgar score on the 14th (Rs = 0.45) and 35th days (Rs = 0.43). In isolation, the RMSSD and Apgar score showed a positive correlation on Day 35 (Rs = 0.41).

In the frequency domain, on Day 1 of life, there was a negative correlation between the LF band and the LF/HF ratio versus the Apgar score (Rs = 0.46), and there was a positive correlation of the HF and Apgar score (Rs = 0.46). On Day 7 of life, again, there was a negative correlation of the LF band (Rs = 0.38) with the LF/HF ratio (Rs = 0.37) and an inverse correlation with the HF band (Rs = 0.37), as shown in Table 8.

For neonatal reflexes, in both groups, there was a correlation with one of the time domain indices on Day 14 of life. As shown for the Apgar score, at this time, there was a positive correlation (*p* < 0.05) between neonatal reflexes and the pNN50 (Rs = 0.53). In the frequency domain, on Day 1 of life, there was a negative correlation between the LF band and the LF/HF ratio versus reflexes (Rs = 0.40) and a positive correlation with the HF (Rs = 0.40).

## 5. Discussion

In the assessment at birth, newborns in the EG had a higher HR than those in the CG because at birth, they had an HR < 180 bpm on auscultation, characterizing neonatal bradycardia. This finding corroborates the data found in other studies because during labor, uterine contractions induce a reduction in blood flow from the mother to the fetus, reducing the partial pressure of oxygen in fetal blood and prolonging physiological hypoxia [29,30]. However, dystocia deliveries requiring surgical intervention lead to neonatal respiratory depression both by fetal asphyxia and by the use of anesthetic medications, which prevent or delay the onset of breathing, exacerbating hypoxemia [23,31]. The consequence of neonatal hypoxia is a decrease in HR [23,32,33].

These differences in the HR and R-R intervals between the groups remained until Day 14, with a progressive increase in HR and a subsequent decrease, and were assumed to be a possible indicator of the development of the influence of the ANS on the heart. It is noteworthy that in the assessment of HR on auscultation, there was an increase in HR in the EG until the 3rd week of life. The cesarean group presented a higher HR peak only until the 7th day of life.

These results showed that newborns from the EG had better physiological adaptation to neonatal development than newborns from the CG. Due to cardiovascular peculiarities at this stage, a high HR is compensatory in neonates of different species [34]. Neonates have a low-pressure circulatory system and lower peripheral vascular resistance than adults. They have lower inotropic capacity, lower compliance, and lower ejection volume values [35,36,37]. As a result, the force of contraction cannot be increased, and the cardiac output depends exclusively on the HR [38].

In this study, in the evaluation of HRV indices, it was observed that the CG presented lower means in the analysis of indices in the time domain (SDNN, RMSSD and pNN50) compared to the EG throughout the first 35 days of life. Thus, at most of the time points evaluated, there were fewer heart rate fluctuations in the CG, and therefore, the group had lower HRV. These differences between the groups suggest that there was greater parasympathetic activity in the EG than in the CG. There was also a positive correlation between these indices and the Apgar score. These results indicated that since the EG had higher vitality at birth, the HRV indices were higher. Therefore, the Apgar score and HRV indices may serve as predictive factors of neonatal viability and possibly predict pathological processes.

Similar to what has been observed in human and animal studies [39,40], in the frequency domain assessment in this study, the LF band was higher than the HF band at all time points in the CG and at most time points in the EG, demonstrating greater sympathetic activity in this neonatal phase. The only times when the LF band was lower and the HF band was higher in the EG were on Days 1 and 7. On these days, there was a difference between the groups in the frequency domain indices, which may indicate greater parasympathetic predominance in the EG than in the CG. The correlation of the LF, LF/HF ratio and HF with the Apgar score showed that the higher the score was, the greater the parasympathetic action and autonomic modulation in the EG compared to the CG.

Since heart rate variability translates into the autonomic and humoral regulation of HR, autonomic balance is directly influenced by the parasympathetic system in healthy dogs. Since the newborns in the CG had dystocia, in their deliveries, there was a prolonged duration of hypoxemia, leading to lower vitality at birth, which can cause myocardial damage [10]. In a study performed by Pereira et al. (2021) [10] evaluating the biomarker of myocardial injury troponin I in neonates with hypoxemia, it was observed that neonates delivered by emergency cesarean section with prolonged asphyxia had higher levels of troponin I in the blood. Thus, it is necessary to measure this parameter in future studies.

It is important to emphasize that for neonates younger than 14 days old, the use of atropine in resuscitation maneuvers is not recommended since neonatal bradycardia is not mediated by the vagal pathway and the sympathetic nervous system is immature in this phase [23,24]. However, in the present study, it was assumed that there was some degree of vagal action in this period. The results obtained for the indices in the frequency domain before 14 days of life, with HF values higher than those found for the LF, indicated some level of parasympathetic action in the group of full-term neonates. This result is important for future studies to better understand the early influence of the ANS on the heart.

In the evaluations of frequency domain indices and physiological parameters, increases in the HR and T°C were observed concomitantly until Day 21 in both groups. Simultaneously, there was a gradual increase in the LF band in this period, also reaching a maximum peak between Days 14 and 21, assuming a possible increase in sympathetic tone in this phase. These data are relevant because neonates depend on room temperature for the maintenance of body temperature due to their inability to thermoregulate [37]. Another mechanism used for thermogenesis is the breakdown of brown fat [23,38]. This lipolysis mechanism is used until the third week of life [41,42]. Since the activation of brown fat breakdown is primarily driven by the sympathetic nervous system [43], it is believed that there was an increase in the LF band in this period as a physiological response to the increased T°C.

In this study, it was evident that the majority of newborns in the CG were brachycephalic. Due to the popularization of these breeds, especially the Bulldog, there has been a parallel increase in the incidence of various diseases affecting this breed [44]. Canine dystocia is an emergency and has a high prevalence in breeds such as the French Bulldog, Boston Terrier, Chihuahuas and Pugs [45]. Thus, it is assumed that there was a greater number of newborns with dystocia among the bulldogs in this study due to their racial predisposition to emergency cesarean sections [44,45,46,47]. These breeds have a reduced pelvic size, as well as a short pelvic canal and a narrower pelvis when compared to non-brachycephalic breeds [48]. Other studies have shown that Bulldogs are the breed most predisposed to dystocia, with uterine inertia being the 3rd most common cause of emergency cesarean sections [45].

It should be noted that the Apgar score is essential for all puppies at birth, but it is even more important for brachycephalic puppies. These breeds have a lower degree of vitality after birth by cesarean section, increasing the risk of mortality [31]. In addition, small breeds have better survival prospects compared to larger breeds [49], making careful assessment of these animals essential.

Currently, there is a high mortality rate of newborns, who often die only a few hours after the appearance of clinical signs. Thus, the detection of predictive factors of the risk of neonatal death, such as birth weight and the Apgar score, is fundamental [50]. In the present study, it was observed that there were differences between the groups in the HRV indices, with correlations at various times with the Apgar score, thus being a method of predicting pathological changes and neonatal death.

The limitations in this study were the containment of the neonates within five minutes so that there was no interference in the electrocardiographic tracing. Because they were neonates and because of contact with alcohol, these animals were reactive at the beginning of the examination, and it was necessary to wait for them to relax.

Thus, equipment that experiences less interference is necessary. Another limitation was that, initially, there was an attempt to place a frequency meter on newborns, but due to the lack of thermoregulation at this stage and, therefore, the impossibility of trichotomy of the region, this method of evaluation was not possible. Given the small number of newborns, further studies are needed to confirm such results. Similarly, it is important, in subsequent research, to compare emergency and elective cesarean sections in brachycephalic breeds.

## 6. Conclusions

Heart rate variability indices and the Apgar score are noninvasive methods that can serve as predictive factors of neonatal vitality. In the present study, both groups showed changes in sympathovagal balance throughout the first 35 days of life, which were more significant in the EG.

Factors that prolong the duration of labor and the use of anesthetic agents in emergency cesarean sections lead to a higher risk of neonatal depression when compared to eutocic vaginal delivery. Therefore, newborns from the CG had lower HRV and, therefore, a greater delay in the influence of the ANS on the heart when facing hypoxia and lower viability at birth.

Further studies are needed for long-term follow-up of animals with lower vitality to assess the behavior of the ANS and to verify whether they will present any pathological processes. The study of other physical factors, such as baroreceptor activity, systemic arterial pressure, and systolic function, may also contribute to the evaluation of cardiovascular homeostasis in the neonatal period.

## Figures and Tables

**Table 1:** Modified Apgar score for newborn dogs [20].

Parameter	Weak (0 Score)	Moderate (1 Score)	Normal (2 Score)
Heart rate	<180 bpm	180 a 220 bpm	>220 bpm
Respiratory rate	No crying/<6 mpm	Mild crying/6 to 15 mpm	Crying/>15 mpm
Reflex irritability	Absent	Grimace	Vigorous
Motility	Flaccid	Some flexions	Active motion
Mucus color	Cyanotic	Pale	Pink

**Table 2:** The neonatal viability reflexes [27].

Parameter	Weak (0 Score)	Moderate (1 Score)	Normal (2 Score)
Suckling	Absent	Weak (>three suckle/minute)	Strong (five suckle/minute)
Rooting	Absent	Slow muzzle fitting inside the circle	Immediate muzzle fitting within the circle
Righting reflexes	Absent (continues in initial position)	Slow body repositioning	Fast body repositioning

**Table 3:** HRV indices in the domain of time and frequency, their meaning and their performance in the autonomous system (Adapted from VANDERLEI et al., 2009) [1].

Time of Domain	Meaning	Autonomous Activity
SDNN	Standard deviation of the mean of all R-R intervals expressed in ms	Sympathetic and parasympathetic activity
rMSSD	Square root of the squared mean of the differences between R-R intervals expressed in ms	Parasympathetic activity
pNN50	Percentage of adjacent R-R intervals with duration difference greater than 50 ms	Parasympathetic activity
**Time of frequency**		
LF (nu)	Low Frequency 0.04 and 0.15 Hz	Sympathetic (predominantly) and parasympathetic
HF (nu)	High Frequency 0.15 to 0.4 Hz	Parasympathetic activity
LF/HF	LF/HF ratio	Simpatovagal tonus

**Table 4:** Mean and standard deviation of the physiological parameters evaluated and statistical significance between times points and groups.

Age	HR (bpm)		RR (mpm)		T°C		Weight (Grams)	
	EG	CG	EG	CG	EG	CG	EG	CG
Birth	196.2 ± 19.2 ^Aa^	160.8 ± 15.7 ^Aa^	23.2 ± 5.1 ^Aa^	25.2 ± 9.8 ^Aa^	33.6 ± 1.6 ^Aa^	33.4 ± 1.0 ^Aa^	126.7 ± 37.4 ^Aa^	261.2 ± 84.0 ^Aa^
(T0)	**(*p* = 0.0000** **)						**(*p* = 0.0000** *)	
1 day	207.3 ± 24.3 ^Ab^	219.2 ± 25.4 ^Aa^	26.2 ± 6.4 ^Ab^	31.7 ± 7.9 ^Ab^	35.7 ± 1.1 ^Ab^	36.3 ± 1.0 ^Ab^	138.0 ± 40.5 ^Ab^	278.6 ± 82.0 ^Ab^
(T1)			**(*p* = 0.0112** *)				**(*p* = 0.0000** *)	
7 days	207.7 ± 18.5 ^Ac^	236.8 ± 15.9 ^Aab^	32.6 ± 11.8 ^A^	40.8 ± 18.0 ^A^	36.9 ± 1.2 ^Ac^	37.1 ± 0.2 ^Ac^	249.0 ± 82.6 ^Ac^	492.0 ± 190.6 ^Ac^
(T2)	**(*p* = 0.0001** **)						**(*p* = 0.0000** *)	
14 days	223.8 ± 14.5 ^A^	218.9 ± 26.7 ^Aa^	31.7 ± 10.9 ^A^	46.6 ± 14.3 ^Aab^	37.0 ± 0.2 ^Ad^	37.2 ± 0.2 ^Aad^	417.4 ± 155.8 ^Aad^	923.4 ± 261.5 ^Aad^
(T3)			**(*p* = 0.0018** *)				**(*p* = 0.0000** *)	
21 days	235 ± 16.4 ^Aabcd^	222.5 ± 22.0 ^Aa^	28.3 ± 6.4 ^A^	42.4 ± 14.7 ^Aa^	37.4 ± 0.3 ^Aab^	37.5 ± 0.2 ^Aab^	606.2 ± 163.8 ^Aab^	1250.0 ± 392.6 ^Aab^
(T4)			**(*p* = 0.0016** *)		**(*p* = 0.0303** **)		**(*p* = 0.0000** *)	
28 days	200.2 ± 23.6 ^Ad^	207.3 ± 22.4 ^Aa^	39.7 ± 8.2 ^Aab^	44.8 ± 13.2 ^Aa^	37.6 ± 0.2 ^Aabd^	37.9 ± 0.3 ^Aabcd^	732.9 ± 182.7 ^Aabc^	1659.2 ± 478.1 ^Aabc^
(T5)					**(*p* = 0.0022** **)		**(*p* = 0.0000** *)	
35 days	202.4 ± 11.7 ^Ad^	194.4 ± 20.0 ^Ab^	37.4± 11.7 ^Aab^	41.0 ± 11.1 ^Aa^	37.7 ± 0.3 ^Aabcd^	38.1 ± 0.3 ^Aabcd^	876.5 ± 304.2 ^Aabcd^	2005.8 ± 477.3 ^Aabcd^
(T6)							(*p* = 0.0000 *)	

Equal lowercase letters overwritten in the same column differ between time points (*p* < 0.05). Isolated uppercase letters do not differ statistically (*p* > 0.05). * Mann-Whitney test; ** *t* test for independent samples (Equivalent Variances); Bold numbers describe significant *p* value between groups.

**Table 5:** Mean and standard deviation of the indices in the time domain (HR and R-R interval) and statistical significance between time points and groups.

Age	HR (bpm)		R-R Interval (ms)	
	EG	CG	EG	CG
Birth	224.9 ± 35.6 ^A^	193.7 ± 23.1 ^Aa^	275.2 ± 58.1 ^A^	314.2 ± 41.2 ^Aa^
(T0)	**(*p* = 0.0024** *)		**(*p* = 0.0024** *)	
1 day	251.4 ± 25.8 ^A^	232.6 ± 20.7 ^Aa^	241.2 ± 26.2 ^A^	259.3 ± 22.9 ^Aa^
(T1)	**(*p* = 0.0367** **)		**(*p* = 0.0549** **)	
7 days	263.0 ± 25.0 ^Aa^	238.7 ± 17.4 ^Aa^	230.0 ± 22.8 ^Aa^	252.6 ± 18.2 ^Aa^
(T2)	**(*p* = 0.0045** **)		**(*p* = 0.0057** **)	
14 days	261.8 ± 34.1 ^Ab^	238.3 ± 14.9 ^Aa^	233.1 ± 34.8 ^Ab^	253.3 ± 15.8 ^Aa^
(T3)	**(*p* = 0.0247** ***)		**(*p* = 0.0237** *)	
21 days	256.8 ± 39.0 ^Ac^	233.5 ± 23.8 ^Aa^	239.4 ± 40.1 ^Ac^	259.6 ± 29.0 ^Aa^
(T4)				
28 days	221.8 ± 24.1 ^Aabc^	221.6 ± 24.6 ^A^	274.0 ± 32.4 ^Aabc^	274.0 ± 31.6 ^A^
(T5)				
35 days	209.8 ± 25.7 ^Aabc^	217.40 ± 24.19 ^A^	290.1 ± 37.0 ^Aabc^	279.0 ± 30.4 ^A^
(T6)				

Equal lowercase letters overwritten in the same column differ between time points (*p* < 0.05). Isolated uppercase letters do not differ statistically (*p* > 0.05). * Mann-Whitney test; ** Test t for independent samples (Equivalent Variances); *** *t* test for independent samples (Different variances); Bold numbers describe significant *p* value between groups.

**Table 6:** Mean and standard deviation of the indices in the time domain (SDNN, RMSSD and pNN50) and statistical significance between time points and groups.

Age	SDNN (ms)		RMSSD (ms)		pNN50 (%)	
	EG	CG	EG	CG	EG	CG
Birth	9.2 ± 4.6 ^Aa^	10.8 ± 4.7 ^A^	10.6 ± 3.7 ^Aa^	12.7 ± 4.2 ^A^	0.3 ± 0.4 ^A^	0.4 ± 0.5 ^A^
(T0)						
1 day	12.5 ± 5.2 ^Ab^	10.3 ± 3.5 ^Aa^	13.0 ± 5.5 ^A^	11.5 ± 5.3 ^Aa^	0.9 ± 1.8 ^A^	0.2 ± 0.2 ^A^
(T1)						
7 days	11.9 ± 5.6 ^Ac^	12.1 ± 2.7 ^A^	12.0 ± 4.9 ^Ab^	13.0 ± 4.5 ^A^	0.5 ± 0.8 ^A^	0.4 ± 0.7 ^A^
(T2)						
14 days	13.3 ± 5.0 ^A^	10.9 ± 3.7 ^A^	13.1 ± 4.6 ^A^	11.5 ± 4.5 ^A^	0.5 ± 0.4 ^A^	0.1 ± 0.2 ^A^
(T3)					(*p* = 0.0014 *)	
21 days	13.8 ± 7.3 ^Ad^	12.0 ± 4.5 ^A^	14.1 ± 7.0 ^A^	12.4 ± 4.8 ^A^	0.8 ± 1.1 ^A^	0.2 ± 0.3 ^A^
(T4)						
28 days	19.5 ± 3.9 ^Aabcd^	14.9 ± 3.1 ^Aa^	17.4 ± 4.0 ^A^	16.0 ± 4.2 ^Aa^	1.2 ± 1.8 ^A^	0.5 ± 0.5 ^A^
(T5)	(*p* = 0.0016 **)					
35 days	20.5 ± 5.2 ^Aac^	13.8 ± 4.3 ^A^	21.3 ± 7.7 ^Aab^	14.7 ± 4.5 ^A^	3.2 ± 5.3 ^A^	0.6 ± 1.0 ^A^
(T6)	(*p* = 0.0007 **)		(*p* = 0.0136 *)		(*p* = 0.0076 *)	

Equal lowercase letters overwritten in the same column differ between time points (*p* < 0.05). Isolated uppercase letters do not differ statistically (*p* > 0.05). * Mann-Whitney test; ** Test t for independent samples (Equivalent Variances); Bold numbers describe significant *p* value between groups.

**Table 7:** Mean and standard deviation of the indices in the frequency domain (LF, HF and LF/HF) and statistical significance between moments and groups.

Age	LF (nu)		HF (nu)		LF/HF	
	EG	CG	EG	CG	EG	CG
Birth	51.3 ± 13.9 ^A^	58.5 ± 20.6 ^A^	47.8 ± 13.8 ^A^	41.3 ± 20.6 ^A^	1.2 ± 0.6 ^A^	2.1 ± 1.8 ^A^
(T0)						
1 day	45.8 ± 12.1 ^A^	63.3 ± 14.5 ^A^	54.0 ± 12.1 ^Aa^	36.5 ± 14.4 ^A^	0.9 ± 0.4 ^Aa^	2.1 ± 1.2 ^A^
(T1)	(*p* = 0.0013 **)		(*p* = 0.0012 **)		(*p* = 0.0025 ***)	
7 days	49.6 ± 15.4 ^A^	64.4 ± 13.7 ^A^	50.1 ± 15.3 ^A^	35.4 ± 13.6 ^A^	1.2 ± 0.7 ^A^	2.3 ± 1.5 ^A^
(T2)	(*p* = 0.0099 **)		(*p* = 0.0098 **)		(*p* = 0.0202 *)	
14 days	58.3 ± 13.7 ^A^	62.6 ± 16.0 ^A^	41.5 ± 13.6 ^A^	37.3 ± 15.8 ^A^	1.8 ± 1.5 ^A^	2.1 ± 1.1 ^A^
(T3)						
21 days	59.1 ± 16.2 ^A^	61.4 ± 11.0 ^A^	40.5 ± 16.1 ^A^	38.4 ± 10.9 ^A^	1.9 ± 1.4 ^A^	1.8 ± 0.8 ^A^
(T4)						
28 days	57.7 ± 18.4 ^A^	52.0 ± 13.2 ^A^	38.1 ± 14.2 ^Aa^	47.7 ± 13.1 ^A^	1.9 ± 0.8 ^Aa^	1.3 ± 1.1 ^A^
(T5)			(*p* = 0.0209 *)		(*p* = 0.0209 *)	
35 days	55.7 ± 15.8 ^A^	57.8 ± 7.9 ^A^	44.0 ± 15.7 ^A^	42.0 ± 7.9 ^A^	1.5 ± 1.1 ^A^	1.4 ± 0.4 ^A^
(T6)						

Equal lowercase letters overwritten in the same column differ between time points (*p* < 0.05). Isolated uppercase letters do not differ statistically (*p* > 0.05). * Mann-Whitney test; ** Test t for independent samples (Equivalent Variances); *** *t* test for independent samples (Different variances); Bold numbers describe significant *p* value between groups.

**Table 8:** Correlation of HRV indices in the time domain with Apgar score.

HRV Indices	Correlation	Times	Coefficient (Rs)
SDNN (ms)	Positive	T5 ^a^; T6 ^b^	0.44 ^a^; 0.49 ^b^
RMSSD (ms)	Positive	T6 ^a^	0.41 ^a^
pNN50 (%)	Positive	T3 ^a^; T6 ^b^	0.45 ^a^; 0.43 ^b^
LF (nu)	Negative	T1 ^a^; T2 ^b^	0.46 ^a^; 0.38 ^b^
HF (nu)	Positive	T1 ^a^; T2 ^b^	0.46 ^a^; 0.37 ^b^
LF/HF (nu)	Negative	T1 ^a^; T2 ^b^	0.46 ^a^; 0.37 ^b^

Equal lowercase letters overwritten on the same line refer to their respective time points.

**Table 9:** HR correlation and R-R interval with Apgar score.

	Correlation	Times	Coefficient (Rs)
HR	Positive	T0 ^a^; T2 ^b^; T3 ^c^	0.39 ^a^; 0.37 ^b^; 0.43 ^c^
R-R Interval	Negative	T0 ^a^; T2 ^b^; T3 ^c^	0.39 ^a^; 0.37 ^b^; 0.43 ^c^

Equal lowercase letters overwritten on the same line refer to their respective time points.

## Data Availability

The raw data supporting the conclusions of this article will be made available by the authors upon request.
